# Organoid cultures recapitulate esophageal adenocarcinoma heterogeneity providing a model for clonality studies and precision therapeutics

**DOI:** 10.1038/s41467-018-05190-9

**Published:** 2018-07-30

**Authors:** Xiaodun Li, Hayley E. Francies, Maria Secrier, Juliane Perner, Ahmad Miremadi, Núria Galeano-Dalmau, William J. Barendt, Laura Letchford, Genevieve M. Leyden, Emma K. Goffin, Andrew Barthorpe, Howard Lightfoot, Elisabeth Chen, James Gilbert, Ayesha Noorani, Ginny Devonshire, Lawrence Bower, Amber Grantham, Shona MacRae, Nicola Grehan, David C. Wedge, Rebecca C. Fitzgerald, Mathew J. Garnett

**Affiliations:** 1 MRC Cancer Unit, University of Cambridge, Cambridge, CB2 0XZ UK; 2 Wellcome Sanger Institute, Hinxton, Cambridge, CB10 1SA UK; 3 Cancer Research UK Cambridge Institute, Cambridge, CB2 0RE UK; 4 Oncology IMED, AstraZeneca, Chesterford, Cambridge, CB10 1XL UK; 5 Cambridge University Hospitals NHS Trust, Cambridge, CB2 0QQ UK; 6 Big Data Institute, University of Oxford, Oxford, OX3 7LF UK; 7 Oxford NIHR Biomedical Research Centre, Oxford, OX4 2PG UK

## Abstract

Esophageal adenocarcinoma (EAC) incidence is increasing while 5-year survival rates remain less than 15%. A lack of experimental models has hampered progress. We have generated clinically annotated EAC organoid cultures that recapitulate the morphology, genomic, and transcriptomic landscape of the primary tumor including point mutations, copy number alterations, and mutational signatures. Karyotyping of organoid cultures has confirmed polyclonality reflecting the clonal architecture of the primary tumor. Furthermore, subclones underwent clonal selection associated with driver gene status. Medium throughput drug sensitivity testing demonstrates the potential of targeting receptor tyrosine kinases and downstream mediators. EAC organoid cultures provide a pre-clinical tool for studies of clonal evolution and precision therapeutics.

## Abstract (web summary)

Esophageal adenocarcinoma (EAC) has a poor 5-year survival rate and lacks robust preclinical models for use in research. Here, the authors show that newly derived organoids recapitulate the transcriptomic, genetic, and morphological landscape of the primary EAC tumors and provide a platform to test drug sensitivity and study tumor clonality.

## Introduction

Esophageal cancer is the eighth most common cancer globally with significant geographical variation in incidence^1^. There are two main histological subtypes, esophageal squamous cell carcinoma (ESCC) and esophageal adenocarcinoma (EAC), the latter has become the dominant subtype in western countries over the past 30 years with a marked increase in tumors occurring around the gastro-esophageal junction. Gastroesophageal reflux is the best documented risk factor for adenocarcinoma which can gradually evolve from the pre-malignant condition Barrett’s esophagus^2^. EAC typically presents de novo, at an advanced stage, and has a poor overall patient survival rates with <15% surviving more than 5 years. With improved multimodality staging methods involving PET-CT and endoscopic ultrasound, there is now a more stringent selection for those patients being treated on a curative pathway. Curative therapy, offered to approximately 55% of patients^3^, generally involves neoadjuvant oncological therapy followed by surgery. The basic regime is consistent while there are regional variations in the chemotherapy regime and whether or not radiotherapy is given; however, the results are consistent with a 5-year survival rate of around 40% in clinical trials^4,5^. Targeted therapy has lagged behind that of other cancers in view of the disappointing trial data for receptor tyrosine kinases (RTKs) in particular. At present, inhibitors of HER2 and VEGFR2 are the only licensed drugs by the FDA for this disease, and they are used as second line therapy for metastatic disease^6,7^. The major obstacles for introducing new therapy approaches have  been a lack of understanding of the molecular genetic drivers of EAC, increasing evidence for a high degree of intra- and inter-tumor heterogeneity, and a lack of physiological model systems for testing hypotheses including those related to clonal evolution and new therapies.

Recent sequencing studies (exome and whole genome) have shown that EAC is a cancer with a high mutation burden^8^, a preponderance of copy number alterations and large-scale chromosomal rearrangements, and the lack of clear recurrent driver genes apart from *TP53*^9–12^. Our recent study based on whole genome sequencing (WGS) data for over 100 cases highlighted: genomic catastrophes (such as chromothripsis and kataegis) in around 30% of cases; almost ubiquitous co-amplification of RTK and disruption of RTK downstream targets; and occurrence of six mutational signatures which provide the potential for patients to be classified according to their dominant signature for therapy decisions^13^.

One bottleneck for esophageal cancer research is a lack of model systems reflecting the primary disease with which to study pathogenesis and sensitivity to therapy^14^. A handful of two-dimensional (2D) cell lines remain the most common in vitro research model and unfortunately most of these lines do not have genomic characterization of the primary from which they were derived and no germline data exist in order to filter out the inherited SNPs^15^. Although the mutational profile of these lines does recapitulate EAC to some extent, the mutational signatures are not representative following in vitro culture over multiple passages^15,16^. Mouse models have been challenging since there is no clear genetic driver akin to *APC* in colon or *KRAS* in pancreatic cancer. Genetically engineered mouse models of Barrett’s have been developed by depleting *p63* or overexpressing IL-1β; however, the mice in the former model do not survive into maturity, and EAC tumors were found in less than 20% mice in the latter model^17,18^. Physiological reflux models have proven technically challenging to achieve in mice due to anatomical differences and the high mortality associated with surgical reflux^14,19^. Hence, there is a high demand from the research community to develop a bank of patient-derived in vitro models that accurately mirror the molecular heterogeneity of clinical EAC.

Advances in the understanding of primary cell niche factors and regulation of signaling pathways to maintain long-term, three-dimensional (3D) ex vivo culture models have provided the breakthrough required to generate primary models from multiple human organs including the GI tract^20–22^. The organoid model system has the capability to overcome limitations of the existing esophageal models by virtue of its stable culture characteristics, flexible manipulation, and faithful recapitulation of the physiological properties of the primary tissue including tissue heterogeneity. Organoid cultures have recently been exploited to study stem cell biology, genomics, disease pathogenesis, and cancer therapy^23^. Some initial investigation was made to generate organoids from Barrett’s esophagus. However, only a small number of cultures were successful, they lack detailed genomic characterization and they have not been made widely available^24^.

The aims of this study are to: (1) Establish a reliable protocol for generating primary EAC organoid cultures with a view to developing a publicly available biobank; (2) Provide a thorough phenotypic and molecular characterization of EAC organoid cultures including karyotyping, genomic profiling, cell kinetics, polarity, and clonality analysis with patient-matched tumor tissues; (3) Evaluate the clonality of EAC organoid cultures and the sub-clonal evolution over time; (4) Demonstrate the feasibility of moderate throughput drug screening to identify new therapeutic targets and precision medicine strategies.

## Results

### Establishment of EAC organoid cultures

Freshly resected EAC tissue samples were collected for organoid derivation from esophagectomy; one tissue sample per patient was utilized. Organoid cultures were successfully established from 10 patients with an overall efficiency of 31% (10 of 32 samples; Table 1), consistent with reported rates for derivation from advanced cancers^25^. Nine of 10 successfully derived cultures were passaged at least 25 times and grew for over 6 months, while CAM298 stopped growing. Histopathological assessment of the primary tumor of CAM298 found this to be the only tumor that was well-differentiated and responsive to neoadjuvant chemotherapy (Table 1). The doubling time of the different organoid cultures was variable, ranging from 18 to 30 hours. The culture conditions were optimized for glandular epithelial cell growth^21,22,24^ and thus minimized competitive growth from normal squamous epithelium. The majority of organoid cultures (6 of 10) were derived from chemotherapy-resistant donors (tumor regression grading (TRG) 4 or 5), although 3 organoids were derived from patients who had not received chemotherapy (Table 1). The primary reasons for culture failure were lack of growth from culture initiation (*n* = 11 cultures), infection (*n* = 5), fibroblast overgrowth (*n* = 4), and arrested growth (*n* = 2). Normal gastric tissue taken at esophagectomy was successfully derived as a healthy glandular control tissue. Unfortunately, cultures from pre-malignant Barrett’s esophagus  were unsuccessful.

**Table 1:** Summary table of clinical characteristics of patient donors

ID	Age	Sex	Stage	Chemotherapy regimen	TRG	Differentiation
CAM298	58	M	ypT1bN0M0	ECX	2	Well
CAM388	78	M	ypT2N0M0	ECX	4	Moderate to poor
CAM247	67	M	ypT2N2M0	ECX	4	Moderate
CAM292	66	F	ypT3N1MX	ECX	4	Moderate to poor
CAM296	72	M	ypT3N3MX	ECF	4	Moderate to poor
CAM338	51	F	ypT3N1MX	ECX	5	Moderate to poor
CAM401	77	F	ypT4aN2M0	CF	5	Poor
CAM412	52	F	cT1bN0M0	No chemotherapy	No chemotherapy	Poor
CAM277	80	F	cT3N2M0	No chemotherapy	No chemotherapy	Poor
CAM408	60	M	cT1aN0M0	No chemotherapy	No chemotherapy	Moderate

The tumor regression grade (TRG) was used to describe histopathological response to chemotherapy, whereby 1 indicates complete regression and 5 is defined as no regressive changes. Patients are ranked by TRG

The acronyms for the chemotherapy regimens are: *ECX* Epirubicin, Oxaliplatin, Capecitabine, *ECF* Epirubicin, Cisplatin, 5-Flourouracil, *EOX* Epirubicin, Oxaliplatin, Capecitabine, and *CF* Cisplatin, Fluorouracil

### Histological characterization of EAC organoid cultures

**Fig. 1:**
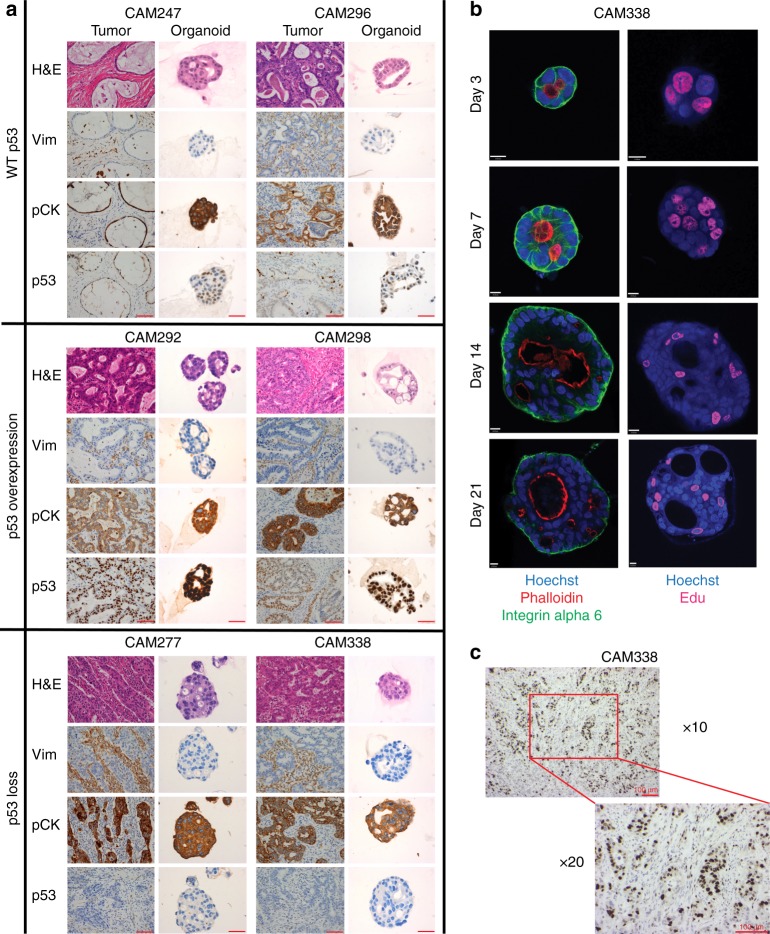
Tumor organoid cultures share histopathological features with patient-matched tissue and disruption of polarity. **a** Representative images of H&E, and IHC of Vim (Vimentin), panCK (pan-cytokeratin), and p53 from primary tissue and patient-matched organoid (40× magnification used for organoid models and 20× for tissues). Images are grouped by p53 expression pattern. Scale bar = 100 μM in primary tissue images and 50 μM in organoid images. **b** Representative whole-mount immunostaining images of tumor-derived organoids at days 3, 7, 14, and 21 after seeding, with anti-phalloidin (selectively stain F-actin, apical marker, Red), integrin α6 (basolateral marker, green), Edu (proliferation marker, pink), and Hoechst (nuclei, blue) as indicated. Scale bar = 10 μM. **c** Representative images of IHC of Ki67 from primary tumor tissue. Scale bar = 100 μM

**Fig. 2:**
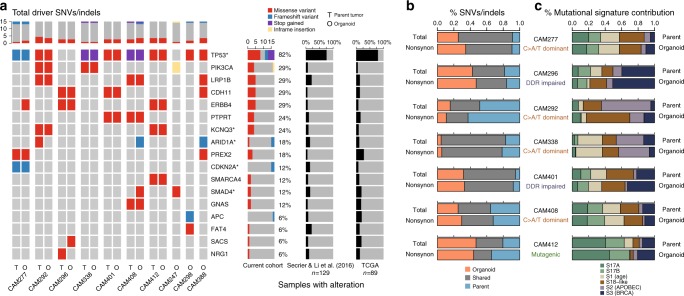
Concordance of driver somatic events, SNV/InDels, and mutational signatures between tumor and matched organoid cultures. **a** Cancer driver genes affected by nonsynonymous SNVs and InDels are highlighted in the derived organoid culture (O) and corresponding patient-matched tumors  (T) where available. Only cancer genes that were also found mutated in at least 5% of the patient cohort (*n* = 129) in the study by Secrier & Li et al.^13^ are displayed, with significantly mutated genes by MutSigCV quoted in the study marked with an asterisk (*). Prevalence of alterations from the analysis of large cohorts of patient tumors (Secrier & Li et al. paper and TCGA (*n* = 89)) are shown on the right. The variant allele frequencies for each mutation are provided. **b** Proportion of shared and unique mutations between patient-matched tumor and organoid culture. **c** Concordance of mutational signature contributions

To verify whether organoids faithfully recapitulated the tumor tissue, we first evaluated the expression of epithelial specific markers. There were a range of Tumor and Lymph Node stages (T, N) and 9  of the cultures showed moderate to poor differentiation as expected from the clinical demographics of the disease (Table 1). Immunohistochemical (IHC) staining was present for pan-cytokeratin with absent vimentin expression confirming an epithelial origin for all organoids (Fig. 1a). Regarding p53 status, 8/10 were mutated, in keeping with the prevalence observed in EAC  patients. Wild-type p53 expression pattern was observed in CAM296 and CAM247, loss in CAM277 (TP53-c.414delC) and CAM338 (TP53-c.574C>T (p.Gln192Ter)), and intense nuclear staining in CAM292 (TP53-c.818G>A (p.Arg273His)) and AH298 (TP53-c.742C>T (p.Arg248Trp)) (Figs. 1a, 2a). In contrast, normal gastric organoid cultures exhibited the morphological characteristics of a normal glandular architecture with wild-type p53 expression pattern (Supplementary Fig. 1a), and wild-type mutational status confirmed by targeted gene sequencing (Supplementary Data 1).

### Disrupted polarity in EAC organoid cultures

Disruption of cell polarity is one of the hallmarks of cancer which is important for tumor initiation and progression. To visualize cell polarity, we evaluated the expression of F-actin and alpha 6 integrin to mark the apical and basal membranes, respectively. EAC-derived organoids had a disordered structure and developed multiple mini-luminal structures from day 3 after seeding, with some inter-patient variation in their degree of complexity and the extent of disruption of polarity (Fig. 1b, Supplementary Fig. 1b). We then examined the distribution of proliferative cells within the organoids. In contrast to the organized proliferative compartment confined to the outer layer in other cancer types^24^, the organoids derived from EAC had proliferative cells present throughout the organoid cell mass, which is in agreement with the diffuse Ki67 staining pattern observed in the primary tumors (Fig. 1c, Supplementary Fig. 1c).

### Genomic characterization of tumor-derived organoids

Whole-genome sequencing was performed to define the genomic landscape of the derived esophageal organoids. The 10 organoids harbored a heterogeneous set of cancer drivers affected by nonsynonymous point mutations or insertions/deletions (InDels), some of which showed a variable pattern of alteration (Fig. 2a). The main EAC drivers reported in the literature to date were observed in this organoid panel and the mutations were consistent between the tumor and the organoid (e.g., *TP53*, *CDKN2A*, *KCNQ3*, *PIK3CA*)^10,13^. This consistency was also observed using the larger cancer gene census from the COSMIC database^26^ (Supplementary Fig. 2a). In general, we observed a higher allele frequency of driver mutations in organoid cultures compared with patient-matched tumors, which is likely to be explained by the pure tumor cellularity in the organoids (Fig. 2a, Supplementary Data 2). Beyond cancer drivers, most of the single nucleotide variants (SNV) and InDels, nonsynonymous or otherwise, were concordant between patient-matched tumor and organoid (Fig. 2b).

Six mutational signatures were observed in the organoid cultures in keeping with EAC tumors^9,10,13^. Generally, the contribution of the dominant signature(s) was consistent between the tumor and organoid culture (Fig. 2c). Furthermore, the cultures reflected the inter-patient heterogeneity reported previously, with 3/10 organoids belonging to the “mutagenic” subtype, 2/10 to the “DDR impaired” subtype, and 5/10 to the “C>A/T dominant” subtype as per the classification proposed in Secrier & Li et al.^13^ (Fig. 2b, Supplementary Table 1, Supplementary Fig. 2b).

**Fig. 3:**
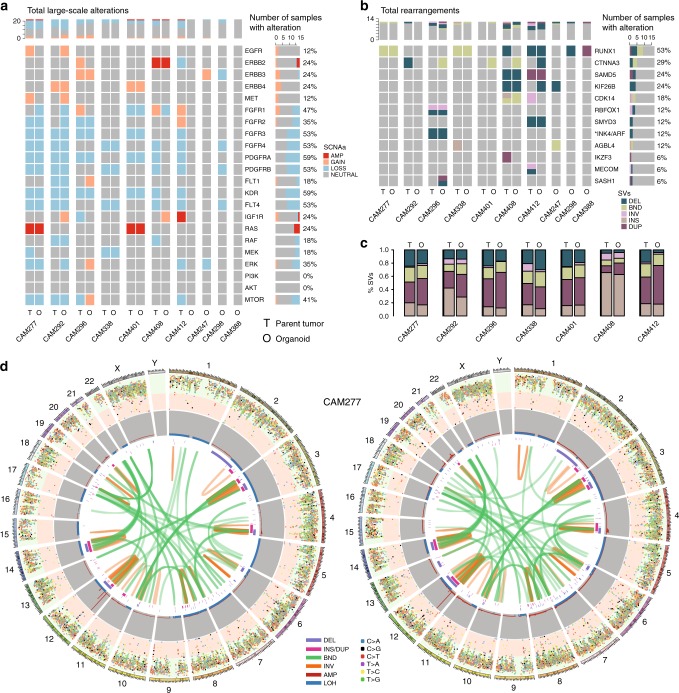
Organoids have a shared genomic landscape with patient-matched tumors. **a** Copy number alterations of selected receptor tyrosine kinases and key downstream pathway mediators are highlighted in tumors and corresponding organoids. **b** Structural variants affecting the top recurrently rearranged genes described in Secrier & Li et al.^13^ and **c** Structural variants are compared between tumor and organoid. **d** Circos plots depicting all mutations (plotted based on inter-mutational distance), copy number changes, and structural variants in the genomes of tumor CAM277 and matched organoid

Large-scale structural alterations showed an even higher degree of concordance than SNVs/InDels, with the overall copy number profiles being largely preserved (Fig. 3a). This consistency was observed for clinically relevant RTKs as well as for other focal or larger genomic segments; for example, amplifications on chromosome 12 in CAM277, CAM401, and CAM296, or on chromosome 17 in CAM408, and chromosome 20 in CAM338 (Fig. 3d, Supplementary Fig. 2c). The patterns of structural rearrangement clearly indicated a common genetic background for each organoid and its respective tumor of origin (Fig. 3b, c, d, Supplementary Fig. 2c). Chromothriptic events on chromosome 16 in CAM296 and the p arm of chromosome 3 in CAM338 were maintained in the respective organoids (Supplementary Fig. 2c).

Some differences between organoid cultures and patient-matched tumors were observed. Additional large segment amplifications were found in the organoid from CAM296, considerably fewer rearrangements in CAM292, as well as loss of ploidy in the CAM292 and CAM296 organoids compared to the tumors. Further, the landscape of SNVs/InDels was more variable than large-scale structural variants (Fig. 2b vs. Fig. 3c). For example, CAM296 had a reduced proportion of shared alterations with its matched tumor, but larger scale patterns such as structural variants or mutational signature composition, which are more likely to have an impact on tumor fitness and development, were generally well preserved.

### Patient-specific gene expression is conserved in organoids

**Fig. 4:**
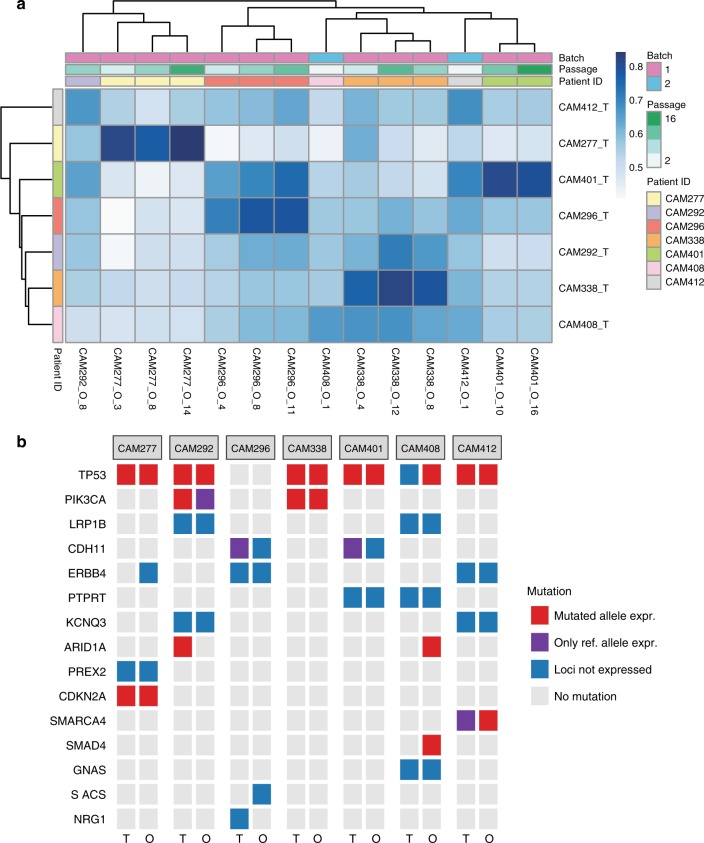
Similarity of global gene expression and expressed mutations between tumors and organoid cultures. **a** Heatmap showing the Pearson correlation coefficient (color key) between tumors (rows) and organoids (columns) based on the normalized counts of tumor- and organoid-specific genes. Dendrograms show the hierarchical clustering based on the complete method and Euclidean pair-wise distance. In some cases, multiple passages from the same organoid culture are included. **b** Mutations as in Fig. 2 colored by whether the mutated allele, the reference allele, or no expression were detected by RNA-seq

Messenger RNA sequencing was performed to quantify phenotypic similarity between the organoid and the tumor EAC samples. Using the Euclidean distance between the overall gene expression profiles, the hierarchical clustering (Supplementary Fig. 3a) indicates that tumors, normal squamous esophagus and organoid samples form separate clusters, with the tumor and normal clusters being more similar to each other than to organoids. This likely reflects the organoid culture environment and the presence of heterogeneous cell types in the primary tumor and normal tissue samples, which are absent from the organoid culture. To identify sets of genes that capture the patient-specific expression profile of the tumors, gene signatures for each patient were defined based on a comparison of tumors and organoids to matched adjacent squamous epithelium and a cross-patient comparison performed (see Methods). The mean correlation coefficient for the pair-wise comparisons of each tumor sample to each organoid was 0.58 (median = 0.57, range = 0.41–0.84), indicating that the expression patterns of tumors and organoids are well correlated (Fig. 4a). Further, for most patients, the highest correlation coefficients were achieved between pairs of organoids and patient-matched tumors. CAM292 and CAM412 organoid samples failed to correlate highly with their counterpart tumor samples, in keeping with the reduced number of shared SNV’s and InDels between these organoid cultures and matched tumors (Fig. 2b).

To elucidate which mutations were expressed, we quantified the expression of SNVs identified from  the WGS analysis and known to be EAC driver genes (Fig. 4b, Supplementary Fig. 3b, c). Genes with SNVs were classified into: not expressed, reference allele expressed, or alternative allele expressed depending on the number of reads at the SNV loci and the variant allele fraction of the alternative allele (see Methods). Consistent with DNA sequencing, there is a high concordance between the patient-matched tumor and the organoid with respect to driver gene mutations expressed at the mRNA level.

### Retention of intra-tumor heterogeneity and clonal dynamics

**Fig. 5:**
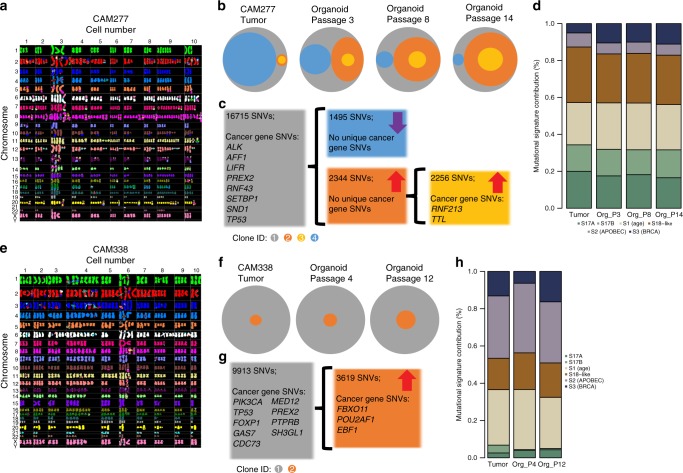
EAC-derived organoids retain intra-tumor heterogeneity. M-FISH spectral karyotype of 10 cells from organoid culture of **a** CAM277 and **e** CAM388. Each chromosome has a unique color. Organoid cultures of **b**, **c**, **d** CAM277 and **f**, **g**, **h** CAM338 were continually cultured and sequenced at different passages to study clonal dynamics. **b**, **f** Segmented Venn diagram represents clonal composition of tumor cells at each time point. The compositions of each clone at different time points are listed in Supplementary Table 2. **c**, **g** Number of total mutations and mutated cancer-related genes from individual subclones. **d**, **h** The percentage contribution for each of the 6 mutational signatures across successive passages. The passage number is depicted as the Org_P value

Given that intra-tumor heterogeneity is a feature of EAC and may impact tumor progression, patient response to therapy, and the emergence of resistance, we asked whether the clonal hierarchy and dynamics are inherited by the derived organoid cultures. To address this question, we first set out to collect direct evidence of the existence of heterogeneity at a single cell level in the organoid culture. Spectral karyotyping (SKY) analysis of organoid cultures revealed that most cells were aneuploid, and all cultures are mixed heterogeneous populations composed of cells with a variety of changes in chromosomal number as well as chromosomal rearrangements (Fig. 5a, e, Supplementary Fig. 4a, b, f). The karyotype of CAM296 (Supplementary Fig. 4b), while heterogeneous, is relatively stable while the karyotype of CAM338 shows evidence of whole-genome duplication (Fig. 5e).

To investigate the clonal structure and dynamics of these cultures, 4 organoid cultures were propagated and samples collected for whole-genome sequencing at multiple passages (up to 16 passages) over a period of approximately 6 months. The organoid genomes were relatively stable with increasing passage with a small increase (<25%) in the number of total mutations and nonsynonymous variants over time, none of which affected cancer drivers (Supplementary Fig. 5a). The proportion of the genome containing amplifications, deletions, or loss of heterozygosity (LOH) regions showed little change over time (Supplementary Fig. 5b). Furthermore, the copy number profile appeared to stay stable between passages (Supplementary Fig. 5c).

To estimate the clone size and dynamics of somatic evolution, we used a Bayesian Dirichelet process to estimate the subclonal architecture at each time point based on the allele frequency of somatic mutations present. This analysis confirmed that the organoid cultures were polyclonal, with representation of all clones found in the matched-primary tumor tissue (Fig. 5b, c, d, f, g, h, Supplementary Fig. 4c, d, e, g, h, i, Supplementary Table 2). Some of these pre-existing subclones were minor in the tumor due to the low cancer cell frequency such as clone 4 in CAM296, and clones 2 and 3 in CAM277.

In line with clonal selection found on engraftment in several PDX model studies^27,28^, the largest changes in subclonal composition were observed at the point of derivation and in the initial passages. In the 4  cultures tested, 2 models (CAM296 and CAM401) showed a rapid evolution then stasis, with minor subclones of the primary tumor becoming almost clonal in the first few passages and remaining dominant throughout propagation (Supplementary Fig. 5c, d, e, g, h, i). In contrast, the clonal evolution of CAM277 and CAM388 was slower with subclonal dynamics ongoing several months after derivation (Fig. 5b, c, d, f, g, h). We also observed that subclones containing somatic nonsynonymous or regulatory mutations in COSMIC cancer driver genes were more likely to expand in culture. For instance, in the trunk of CAM277 (clone 1), which contained 16715 somatic mutations including *ALK* and *TP53*, was fully clonal in the primary tumor and remained clonal in organoid cultures; branch clone (clone 2) and its subclone (clone 3) expanded during organoid propagation. In particular, clone 2 containing somatic mutation of *RNF213* (E3 ubiquitin-protein ligase involving noncanonical WNT signaling pathway^29^), was almost clonal by the most recent passage. Conversely, another branch clone (clone 4), which was nearly clonal in the tumor and lacked driver mutations, gradually decreased in dominance in the derived organoids (Fig. 5c). Similarly, in CAM296, mutated *KRAS* and *BCL6* were found in subclone 4 which became the dominate clone in culture (Supplementary Fig. 5d). Further studies are required to interpret the significance of driver genes in clonal expansion. Interestingly, regardless of the dynamic evolution of subclones within organoid cultures, the six mutational signatures were extremely stable over time, particularly with regard to the dominant mutational signature (Fig. 5d, h and Supplementary Fig. 4e, i).

### Drug sensitivity in EAC organoid cultures

We next evaluated the applicability of EAC organoids grown as 3D cultures for drug sensitivity testing. We tested their sensitivity to 24 anti-cancer compounds using a 7-point half-log dilution series (1000× concentration range), including eight FDA approved drugs and 10 preclinical molecularly targeted agents against key targets and pathways implicated in EAC (listed in Supplementary Data 3)^22,30^. There was a good positive correlation of the area under the dose–response curve (AUC) values across biological replicates (*n* = 2 or 3 replicates; Rs > 0.8) for the entire dataset (Supplementary Fig. 6a). Compounds with overlapping targets had similar activity across the panel of organoid cultures (Rs = 0.92 for PI3K inhibitors, Rs = 0.69 for IGF1R inhibitors, and Rs = 0.53 for EGFR inhibitors, *n* = 9 organoids) (Supplementary Fig. 6b).

Despite on-going clonal evolution, drug responses of individual organoid cultures were consistent over time when comparing sensitivity following prolonged culturing of up to 1 year (Rs ≥ 0.85) (Supplementary Fig. 6c, d). Furthermore, drug sensitivity was similar whether organoid cultures were plated for screening as single cells or already formed multi-cellular organoids (Supplementary Fig. 6e).

**Fig. 6:**
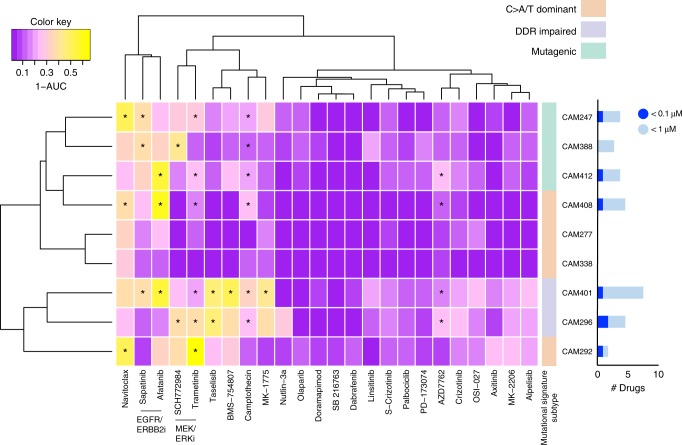
Drug sensitivity profiling of 24 compounds against nine EAC organoids. Organoid cultures are clustered based on their drug sensitivity as measured by 1-AUC values across the drug panel. The drug names are provided at the bottom of the panel and the mutational signature subtype also indicated. Asterisks (*) identify drugs that elicited an IC_50_ below 1 µM while the histogram indicates the number of drugs for each organoid culture eliciting an IC_50_ below 1 or 0.1 μM

Consistent with the heterogeneity of EAC, we observed a range of compound sensitivities across the nine organoid cultures (Fig. 6). CAM277 and CAM338 were resistant to the majority of compounds tested, with all IC_50_ values being greater than the maximum screening concentration (10 μM for the majority of compounds). The organoids with a DDR-impaired signature (CAM401 and CAM296) were sensitive to the greatest number of compounds. For the remaining five organoid cultures, sensitivities could be identified to at least two targeted agents, eliciting an IC_50_ below a drug concentration of 1 or 0.1 μM.

Whilst CAM296 and CAM247 were *TP53* wild-type, only CAM296 demonstrated sensitivity to the MDM2 inhibitor Nutlin-3a (IC_50_ 3.1 μM) while CAM247 failed to respond (IC_50_ 16 μM). The mediator of resistance in CAM247 could not be identified. However, the varying sensitivity of wild-type *TP53* lines is not uncommon^31^. Unsupervised clustering analysis of the cell viability following drug treatment revealed clustering of the ERK inhibitor SCH772984 and the MEK1/2 inhibitor Trametinib, as well clustering of the EGFR-family inhibitors Afatanib and Sapatinib. Notable sensitivities observed were the MEK1/2 inhibitor Trametinib (*n* = 6 of 9 organoids with IC_50_ < 0.1 μM), and with the EGFR/ERBB2 inhibitor Afatinib (*n* = 3 of 9 organoids with IC_50_ < 250 nM).

We additionally examined the sensitivity of eight organoid cultures to standard EAC chemotherapy agents 5-fluoruracil, epirubicin, and cisplatin (Supplementary Fig. 7a, b). The only patient in our panel who responded to chemotherapy (TRG 2) had a well-differentiated organoid which did not proliferate sufficiently for drug testing (CAM298). Six of the organoid cultures (CAM388, CAM296, CAM338, CAM412, CAM277, CAM408) were insensitive to single and combination chemotherapy drugs. Three of these organoid cultures derived from chemo-treated patients (CAM388, CAM296, CAM338) who all had a poor response to therapy (TRG 4 or 5). Two organoid cultures showed some limited sensitivity (CAM247 and CAM401). Overall, the lack of chemotherapy sensitivity for most organoid cultures (6 of 8) was consistent with the poor in vivo response to neo-adjuvant chemotherapy seen in this disease (Table 1).

## Discussion

Here we demonstrate that human EAC organoid cultures can be generated from human tissue retrieved at surgical resection. A panel of 10 organoid cultures was generated with a success rate of 31%. Ninety percent  of these were capable of long-term growth (>6 months) in culture. Our analyses provide insight into the clonality of organoid cultures and the dynamics of tumor subclones over extended in vitro culturing. This renewable resource enriches the  in vitro cell models of EAC and provides the research community with models that have extensive clinical, cellular, and molecular data including germline reference sequence.

The comprehensive characterization of the organoid cultures confirmed that they recapitulated the features of the primary tumors from which they were derived in terms of histology, cancer driver alterations, mutational/copy-number/large-scale genomic rearrangement landscape, mutational signature subtypes, as well as expression profile. Nevertheless, we did observe gene expression signatures and genetic alterations unique to either the tumor or the organoid model for all of the cases. Differences in gene expression are likely due to the distinctive microenvironments between the primary tumor and the organoid culture system. Differences in genetic alteration detection are likely due to the heterogeneity of the tumor of origin causing sampling bias and/or selection of subclones during organoid derivation and propagation. Furthermore, mutations have a higher variant allele fraction in organoid cultures making SNV easier to detect compared to patient tissue (Fig. 2a, Supplementary data 2). For example, in CAM277, a one-nucleotide deletion (Chr9:21972087) of *CDKN2A* was detected in 8  of 33 total reads in the primary tumor; in contrast, it was found in 23 of 23 total reads in the derived organoid culture (Supplementary Data 2). We therefore curated variants called inconsistently between the organoid and the primary, but nevertheless, this could lead to some discordant calls (Fig. 2a). Notably, additional variants identified in organoid cultures do not result in the acquisition of additional EAC specific cancer driver genes absent in the patient-matched tumor. The stability of organoid culture was reflected by the lack of additional cancer driver mutations emerging and consistent mutational signatures following longitudinal culturing.

SKY and clonality analysis confirmed that the organoid cultures contain multiple subclones and these are shared with the patient-matched tumor. The initial derivation of an organoid culture led to the greatest selection pressure on genetic subclones found in the tumor, and consequently the contribution of each subclone in a culture. Nonetheless, all clones observed in the organoid cultures could be traced back to the original tumor. Organoid heterogeneity was maintained during culturing over several months for all organoids tested, although dynamic clonal behavior was observed in some models whereas others cultures were relatively stable. Our observation of ongoing clonal evolution in organoid cultures indicates that these models could provide a facile in vitro experimental system to investigate factors such as the stromal cells, niche-factors and inter-clonal cooperation/competition, and ordering of driver mutations, which could impact on clonal evolution during tumor growth and response to therapy.

Drug sensitivity testing using in vitro cancer cell models, is used extensively during drug development in academia and industry. Consistent with studies in colon^22^, pancreas^32^, liver^33^, and prostate^34^ cancers, our results confirm the tractability of using EAC organoids for testing drug sensitivity. A range of sensitivities to compounds were observed across the panel of EAC cultures, including differential sensitivity to EGFR and MEK inhibitors. This is consistent with the highly heterogeneous nature of EAC and underscores the challenge of developing effective treatments. This study was designed to evaluate the feasibility of drug testing in EAC organoids. A larger collection of EAC models, such as those being generated by an international consortium such as the Human Cancer Models Initiative, will enable the robust association of drug response with molecular markers, and further studies are required to evaluate the in vivo relevance of the drug candidates identified here. Despite evidence for dynamic clonal populations in some organoid cultures, drug sensitivity profiles were generally stable when compared after prolonged periods in culture, indicating that the dominant determinant(s) of sensitivity to a drug are shared by the different subclones. This does not exclude the possibility of rare cells in a culture with differential drug sensitivity, and which can underpin clinical resistance to some therapies.

In general, the organoid cultures were insensitive to standard combinations of EAC chemotherapy agents consistent with patient responses. Indeed, following tumor resection, there is rarely sufficient tumor material available for organoid derivation from patients who respond well to therapy. In the two instances where sensitivity to chemotherapy was observed, this may be due to clonal selection during derivation (as observed for CAM401), tumor evolution in the time between neo-adjuvant chemotherapy and surgery (typically 3 months), the subjectivity of the TRG classification, and differences in the in vivo tumor environment. In the future, these models could be used to test for response to radiotherapy, especially since this is increasingly used as standard therapy, and the inclusion of stromal and immune cells could further expand the utility of these models.

In summary, a panel of EAC organoids has been derived with extensive clinical, genomic, and phenotypic characterization. These models reflect the morphological, functional, and genetic features of the tissue of origin and can now be used to address clinical translational questions including tumor heterogeneity and evolution, as well as providing insights into drug sensitivity.

## Methods

### Ethical approval and sample collection

The study was registered (UKCRNID 8880), approved by the Institutional Ethics Committees (REC 07/H0305/52 and 10/H0305/1), and all participants gave written informed consent as part of the OCCAMS (Oesophageal Clinical And Molecular Stratification) consortium. Samples were obtained from surgical resection. Half of the collected tissue samples from each of the patients were prepared for organoid derivation while the other half were snap-frozen using liquid nitrogen and stored at −80 °C until used for genomic profiling. Blood or normal squamous esophageal samples (sampled at least 5 cm in distance from the tumor) were used as a germline reference. Haematoxylin and Eosin stained frozen tissue sections were reviewed for tumor cellularity by two pathologists independently. Tumor tissue samples only with ≥70% cellularity (primary tumor tissues of CAM247 and CAM298 were lower than this threshold, therefore only primary normal tissue samples were sequenced for organoid genomic characterization) were selected for DNA and RNA extraction using the AllPrep kit (Qiagen) as were normal squamous esophageal tissue samples. DNA was extracted from blood samples using the QIAamp DNA Blood Maxi kit (Qiagen).

### Tumor and normal gastric organoid derivation

Tumor samples underwent multiple washes with PBS before being minced into small pieces using a scalpel and incubated with collagenase II (1.5 mg/ml) for 1–2 h at 37 °C. Following incubation, the mixture was filtered through a 70-μM cell strainer to remove large undigested fragments. The cell suspension was centrifuged at 300–400×*g* for 2 min. The cell pellet was resuspended in PBS and centrifugation repeated. This procedure was repeated twice to remove debris and collagenase.

The isolated cells were re-suspended in 7.5 mg/ml basement membrane matrix (Cultrex BME RGF type 2 (BME-2), Amsbio) supplemented with complete media and plated as 10–15 μl droplets in a 6-well plate. After allowing the BME-2 to polymerize, complete media was added and the cells left at 37 °C.

Complete media: AdDMEM/F12 medium supplemented with HEPES (1×, Invitrogen), Glutamax (1×, Invitrogen), penicillin/streptomycin (1×, Invitrogen), B27 (1×, Invitrogen), Primocin (1 mg/ml, InvivoGen), N-acetyl-L-cysteine (1 mM, Sigma) Wnt3a-conditioned medium (50% v/v, L-WNT3A cell line is available from ATCC), RSPO1-conditioned medium (20% v/v, the cells were kindly provided by Calvin Kuo. A cell line is also available from Trevigen.), recombinant Noggin protein (0.1 μg/ml, Peprotech), epidermal growth factor (EGF, 50 ng/ml, Peprotech), fibroblast growth factor 10 (FGF10, 100 ng/ml, Peprotech), Nicotinamide (10 mM, Sigma), SB202190 (10 μM, Stem Cell Technologies), and A83-01 (0.5 μM, Tocris).

### Organoid culture

Organoid culture medium was refreshed every 2 days. To passage the organoids, BME-2 was disassociated by pipetting. The organoids were collected in a falcon tube and TrypLE (Invitrogen) added before being incubated at 37 °C for approximately 5 min. A vigorous manual shake would ensue before the suspension was centrifuged at 300–400×*g* for 2 min. The remaining cell pellet was re-suspended in 7.5 mg/ml BME-2 supplemented with complete media and plated as 10–15 μl droplets in a 6-well plate. After allowing the BME-2 to polymerize, complete media was added and the cells left at 37 °C.

### Whole-genome sequencing

Snap-frozen tissues and blood samples were prepared for whole-genome sequencing as part of the OCCAMS Consortium ICGC project by Illumina. Paired-end sequencing (100 bp) was performed to a depth of 50× for tumors and 30× for DNA derived from normal squamous esophageal tissue or blood samples. The analysis demonstrated that 94% of the genome was sequenced to at least 8× coverage while achieving a PHRED quality of at least 30 for at least 80% of mapping bases. QC metrics were computed on a per-lane basis using FastQC (http://www.bioinformatics.babraham.ac.uk/projects/fastqc) and in-house tools, enabling the identification of sequence reads that required trimming. DNA extracted from snap-frozen organoid cell pellets was prepared for whole-genome sequencing. Paired-end sequencing (150 bp) was conducted on the Illumina HiSeqX platform at a depth of 30×.

### RNA sequencing

RNA-sequencing of organoids was performed using Illumina TruSeq Stranded mRNA LT Sample Prep Kit Set A and Set B. mRNA was purified using Oligo dT attached magnetic beads and then fragmented using divalent cations. The mRNA fragments were copied into first strand cDNA using random hexamer primers and reverse transcriptase. Second strand synthesis employed DNA polymerase I and RNase H. cDNA fragments underwent end-repair, the addition of a single A base and ligation of indexed adapters. Products were purified and enriched by PCR using Kapa HiFi polymerase prior to quantification and pooling prior to sequencing on an Illumina HiSeq 4000 instrument at 75 bp PE.

### Whole-genome sequencing analysis

Read sequences were mapped to the human reference genome (GRCh37) using Burrows-Wheeler Alignment (BWA) 0.5.9^35^. Duplicates were marked and discarded using Picard 1.105 (http://broadinstitute.github.io/picard). The data underwent an extensive quality assurance process, and quality control metrics and alignment statistics were computed on a per-lane basis. The FastQC package was used to assess the quality score distribution of the sequencing reads and perform trimming if necessary. Somatic mutations and InDels were called using Strelka 1.0.13^36^ and subsequent filtering was applied as described in ref.^13^ including DistanceToAlignmentEndMedia, DistanceToAlignmentEndMAD, LowMapQual, MapQualDiffMedian, VariantMapQualMedian, VariantBaseQualMedian, VariantAlleleCount, VariantAlleleCountControl, StrandBias, Repeat, SNVCluster50, SNVCluster 100). The resulting variants were functionally annotated using Variant Effect Predictor (VEP release 75). In order to aid the detection of variants called in the organoids and missed in the tumor due to allele frequency below the imposed cut-offs (as a result of low purity/subclonal selection) in an automated manner, we combined the BAM files from the tumor and patient-matched organoid culture, and recalled the SNVs and InDels using Strelka on the combined resulting reads with the same filtering cut-offs. This step had the effect of increasing the read count and confidence of calling real variants for the ones that differed between tumor and organoid. Key driver genes were also manually inspected to confirm the pooled call results.

In addition, we calculated allele counts from the BAM files for tumor, organoid, and pooled samples at all positions where a variant was called in any sample for the case. For the pooled BAM files, allele frequencies at some positions were raised above the threshold for consideration by Strelka, and overall the number of variants called by Strelka and passing GATK filters was increased compared with the individual BAM files. For each case, variants that were called from the pooled BAM file, but only called in the tumor or the organoid(s), are considered as present in both the tumor and the organoid(s) if the variant allele is represented in the tumor BAM file and the organoid BAM file(s). Such variants in key driver genes were manually inspected using the IGV tool, to confirm the concordance between the tumor and the organoid(s).

Copy number segmentation was performed using ASCAT-NGS v2.1^37^, using read counts at germline heterozygous positions estimated by GATK 3.2-2^38^. Cut-offs for copy number changes were defined based on the segmental copy number relative to the average ploidy *p*_*s*_ in the sample as follows: amplification: ≥2 × *p*_*s*_; gain: >1.25 × *p*_*s*_; loss: <0.75 × *p*_*s*_, deletion: copy number 0. For selected key genes in pathways downstream of RTKs, the average copy number across all genes in the respective family was calculated (e.g., RAS would summarize the copy number landscape of HRAS, KRAS, and NRAS) and subsequently mapped according to the defined cut-offs. Structural variants were called using Manta^39^. SVs were filtered against a panel of matched tissue normals. We then filtered out structural variant calls overlapping problematic regions such as gaps, satellite sequences, simple repeats >1000 base pairs and extreme read depth regions. We also discarded deletions <1000 base pairs not supported by at least one read, and inversions of <10 kb which are likely to be artefactual. Exposures to the six EAC signatures described in previous study^13^ were inferred using a quadratic programming approach that models the mutational counts in each genome based on the pre-defined mutational probabilities of these signatures, as detailed in ref.^13^. Subsequent assignment of organoids to one of the three mutational signature subtypes was performed based on the most prevalent individual signature contribution in the respective genome, with the exception of S17A and S17B where a summed contribution counted toward assignment to the “mutagenic” signature.

### RNA-seq analysis

Gene expression was quantified using Salmon^40^ in quasi-mapping mode and with parameters −*l* = ISR and −*p* = 10. The Ensembl gene annotation GRCh37.87 was provided. Transcript expression was collapsed to gene-level expression in R using tximport^41^ and providing the transcript-to-gene mapping from the Ensembl gene annotation. Genes with zero counts in all samples were excluded from the analysis. Read counts were normalized and log2-transformed using variance stabilization (vst) as implemented in DESeq2^42^ setting blind = FALSE and taking into account the sample class, which here indicates whether the sample is a tumor, normal squamous esophagus, or organoid.

For quantifying the expression of SNVS, RNA-seq reads were mapped to the GRCh37_g1k genome assembly using STAR^43^. Following GATK’s^38^ best practice on ‘Calling variants in RNAseq’, readgroups were added and duplicates marked using Picard^44^. GATK’s SplitNCigarReads for trimming reads and assigning mapping qualities was applied. The sets of SNVs identified using Strelka on WGS data for all tumor or organoid were merged using vcftools^45^. Reads overlapping any SNV were counted using the ReadCountWalker in gatk-tools^38^. Mutations were called expressed if the read depth was larger or equal 4 and the variant allele frequency was larger than 0. Supplementary Fig. 3a shows the fraction of expressed mutations in expressed loci where an SNV was detected in the WGS analysis. The tumor sample for CAM412 showed much fewer expressed mutations than other samples and variant allele frequencies were low (Supplementary Fig. 3c).

Normalized read counts per gene were used to cluster samples using hierarchical clustering with complete linkage and Euclidean distance. The distance matrix and the clustering was visualized as a heatmap using pheatmap. The clustering indicated that for CAM412 the tumor sample showed an expression pattern more similar to normal samples than to other tumors, and the normal sample showed comparably little similarity to other normals. Together with the observation of few mutations being expressed in the tumor sample, we excluded CAM412 from further analysis due to quality issues.

A detailed comparison of the expression patterns of primary tumor and organoids was performed to see if the organoid resembles the expression profile of the tumor. There are several factors that can confound this analysis. For example, patient samples are a mixture of cancer cells and cells from adjacent tissue, immune cell, or stroma, which are not present in the organoids. Further, organoids are exposed to an artificial environment that is different from the primary tissue. To cope with these effects, the set of genes that were used to compare tumor and organoid samples were preselected as follows. First, only genes that are differentially expressed between normal and tumor samples and that are less expressed in normals (*p* ≤ 0.01, log2-FoldChange ≤ −1) compared to tumors were selected. Second, genes that are generally differentially expressed between tumors and organoids (*p* ≤ 0.01, abs(log2-FoldChange) ≥1) are thought to be the effects of the different environment that organoids are exposed to and were discarded. Finally, for each patient, the genes that are specifically up- or downregulated in each patient’s organoid (1 vs. all comparison, *p* ≤ 0.05 and abs(log2-FoldChange) ≤ 1) and that are among the top-50 genes with highest base mean expression were selected.

### Immunohistochemistry

Paraffin embedded sections of 3.5 μm were stained by a Bond Max autostainer according to the manufacturer’s instruction (Leica Microsystems). Primary antibodies cytokeratin (AE1/AE3, 1:100, Dako), Vimentin (D21H3, 1:100, Cell Signaling Technology), and p53 (D07, 1:50, Leica) were optimized and applied with negative controls.

### Immunofluorescence

Briefly, organoids were fixed with 4% PFA, permeabilized in Triton X-100, quenched with glycine-PBS and then incubated with primary antibody of integrin α6 (1:100, BioLegend) overnight. Organoids were washed and incubated with appropriate secondary antibody along with Phalloidin (Thermo Fisher). Nuclei was stained by Hoechst (Thermo Fisher). Organoids were imaged by confocal microscope TCS SP5 (Leica), Z stacks were taken at 1-μm intervals through organoids, and images were processed by Volocity image analyze software (Perkin Elmer, version 6.3.0).

### Chromosome harvest and multiplex-FISH karyotyping

Cultures were incubated for 3 h with 0.1 μg/ml Karyomax Colcemid (Gibco) before being harvested and dissociated using TrypLE (Gibco). Cells were incubated with buffered hyptonic solution (0.4% KCL in 10 MM HEPES) for 8–12 min at 37 °C. The cells were then fixed and washed in a 6:1 (v/v) methanol:glacial fixatives and stored at −20 °C until use. Harvesting of chromosomes and multiplex-FISH karyotyping was conducted as previously described^46^.

The organoid cultures were incubated for 3 h with 0.1 μg/ml Karyomax Colcemid (Gibco) before being harvested and dissociated using TrypLE (Gibco). Cells were incubated with buffered hypotonic solution (0.4% KCl in 10 MM HEPES) for 8–12 min at 37 °C. The cells were then fixed and washed in a 6:1 (v/v) methanol:glacial fixative and stored at −20 °C until use.

For multiplex-fluorescence in situ hybridization (M-FISH), chromosome-specific DNA libraries were generated from 5000 copies of flow-sorted chromosomes, using GenomePlex Whole Genome Amplification (WGA2) kit (Sigma-Aldrich). Human 24-color painting probe was made following the pooling strategy^47^. Five human chromosome pools were labeled with ATTO 425-, ATTO 488-, CY3-, CY5-, and Texas Red-dUTPs (Jena Bioscience), respectively, using WGA 3 re-amplification kit (Sigma-Aldrich) and home-made dNTP mixtures optimized for the incorporation of the aforementioned labeled dUTPs by Taq polymerase. The labeled products were pooled and sonicated to achieve a size range of 200–1000 bp, optimal for chromosome painting. The sonicated DNA sample was precipitated with ethanol together with human Cot-1 DNA (Invitrogen) and resuspended in a hybridization buffer (50% formamide, 2× SSC, 10% dextran sulfate, 0.5 M phosphate buffer, 1× Denhardt’s solution [pH 7.4]). Metaphase preparations were dropped onto precleaned microscopic slides, followed by fixation in acetone (Sigma-Aldrich) for 10 min and dehydration through an ethanol series (70%, 90%, and 100%). Metaphase spreads on slides were denatured by immersion in an alkaline denaturation solution (0.5 M NaOH, 1.0 M NaCl) for 7–8 min, followed by rinsing in 1 M Tris-HCl (pH 7.4) solution for 3 min, 1× PBS for 3 min, and dehydration through a 70%, 90%, and 100% ethanol series. The M-FISH probe was denatured at 65 °C for 10 min before being applied onto the denatured slides. The hybridization area was sealed with a 22 × 22-mm coverslip and rubber cement. Hybridization was carried out in a 37 °C incubator for two nights. The post-hybridization washes included a 5-min stringent wash in 0.5× SSC at 75 °C, followed by a 5-min rinse in 2× SSC containing 0.05% Tween20 (VWR) and a 2-min rinse in 1× PBS, both at room temperature. Finally, slides were mounted with SlowFade Gold mounting solution containing 4′6-diamidino-2-phenylindole (Invitrogen). Images were visualized on a Zeiss AxioImager D1 fluorescent microscope equipped with narrow band-pass filters for DAPI, DEAC, FITC, CY3, TEXAS RED, and CY5 fluorescence and an ORCA-EA CCD camera (Hamamatsu). M-FISH digital images were captured using the SmartCapture software (Digital Scientific UK) and processed using the SmartType Karyotyper software (Digital Scientific UK). Approximately 20 metaphase chromosomes from each organoid culture were fully karyotyped based on M-FISH classification.

### Clonality analysis

Analysis of clonal dynamics was carried out on four tumors (CAM277, CAM296, CAM338, and CAM401). For each primary tumor and each derived organoid, copy number aberrations, tumor ploidy, and purity were called using the Battenberg algorithm^48^. Briefly, the algorithm phases heterozygous SNPs with use of the 1000 genomes genotypes as a reference panel. The resulting haplotypes are corrected for occasional errors in phasing in regions with low linkage disequilibrium. After segmentation of the resulting b-allele frequency (BAF) values, *t*-tests are performed on the BAFs of each copy number segment to identify whether they correspond to the value resulting from a fully clonal copy number change. If not, the copy number segment is represented as a mixture of two different copy number states, with the fraction of cells bearing each copy number state estimated from the average BAF of the heterozygous SNPs in that segment.

As expected, all organoids had purities estimated by Battenberg of 99–100%, while primary tumors had estimated purities ranging between 33% and 78%. Mutations were clustered to identify subclones, using a previously described Bayesian Dirichlet process^49^. The fraction of cancerous cells represented by each subclone, or ‘cancer cell fraction’ (CCF) in each sample (tumor or organoid) was estimated as the median of the posterior CCF obtained from the Dirichlet process. Quality control of subclonal clusters was performed to filter out clusters that may result from artefacts in mutation or copy number calling, using three metrics: (1) Clusters containing fewer than 1% of all mutations identified within a tumor; (2) Clusters for which the median CCF of the mutations assigned to that cluster differed by more than 0.2 from the CCF output from the Dirichlet process clustering, in one or more samples; (3) Clusters in which more than 50% mutations occurred on the same chromosome. The previously described ‘sum’ and ‘crossing’ rules^50^ were used to determine whether subclones should be nested or disjoint in Fig. 5 and Supplementary Fig. 4.

### Drug screening assays

Thirty-two microliters of an organoid suspension were dispensed into all wells of a 384-well plate containing 8 μl of 7.5 mg/ml BME-2, for a total assay volume of 40 μl. A 7-point half-log dilution series of each compound was dispensed using liquid handling robotics the following day and cell viability assayed using CellTiter-Glo^®^ (Promega) following 6 days of drug incubation^22,30^. Screens were performed in technical duplicate and for 6 of the 9 organoids biological replicates were generated. AUC and IC_50_ values are the mean across all replicates. All screening plates were subjected to a manual inspection of data and a Z-factor score comparing negative and positive control wells calculated (median = 0.58; range (0.1–0.87). Dose–response curves were fitted to the luminescent signal^51^. Compound and screening concentrations are provided in Supplementary Data 3.

Profiling of the organoid models to the chemotherapy agents was conducted similar to that described above. The drug combinations were conducted using an anchored approach, whereby cisplatin and 5-fluorouracil were kept constant at a single concentration of 4 μM and 10 μM, respectively, while a 7-point half-log dilution series of epirubicin (maximum concentration 10 μM) was used.

### Data availability

The RNA and whole-genome DNA sequencing data are available at the European Genome-phenome Archive (EGA) under accession EGAD00001004007. Information to match the sample identifier to the patients and organoids is provided in Supplementary Table 3.

## Electronic supplementary material

Supplementary Information

Description of Additional Supplementary Files

Supplementary Data 1

Supplementary Data 2

Supplementary Data 3
