# Supplementary material for: Organoid cultures recapitulate esophageal adenocarcinoma heterogeneity providing a model for clonality studies and precision therapeutics
Source: Nat Commun. 2018 Jul 30;9:2983. doi: 10.1038/s41467-018-05190-9 (PMC6065407; doi:10.1038/s41467-018-05190-9)
Supplement: Supplementary file 1 — Supplementary Information [file 41467_2018_5190_MOESM1_ESM.pdf]

Organoid cultures recapitulate esophageal adenocarcinoma heterogeneity providing a model for clonality studies and precision therapeutics

Li & Francies et al.

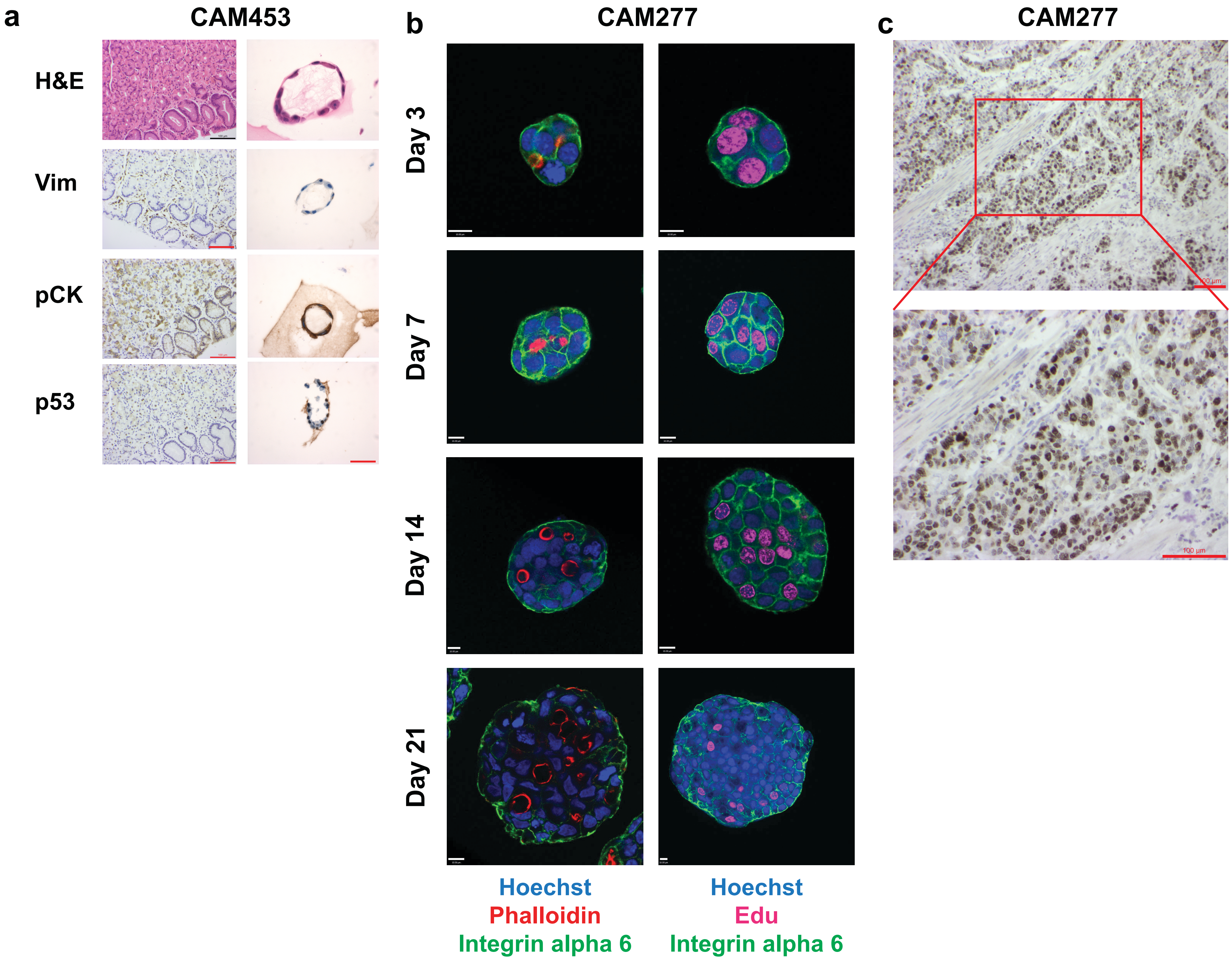

Supplementary Figure 1: Normal gastric organoid cultures share histo-pathological features with tissue and disruption of polarity is observed in tumor derived organoids

(a) Representative images of H&E, and IHC of Vim (Vimentin), panCK (pan-cytokeratin) and p53 from primary tissue and its derived normal gastric organoid (x40 magnification used for organoid models and x20 for tissues). Scale bar =100 µM in primary tissue images and 50 µM in organoid images, (b) Representative whole-mount immunostaining images of CAM277 tumor derived organoids at day 3, 7, 14 and 21 after seeding, with anti-Phalloidin (selectively stain F-actin, apical marker, Red), Intergrin α6 (basolateral marker, green), Edu (proliferation marker, pink) and Hoechst (nuclei, blue) as indicated. Scale bar = 10 µM. (c) Representative images of IHC of Ki67 from primary tumor tissue. Scale bar = 100 µM.

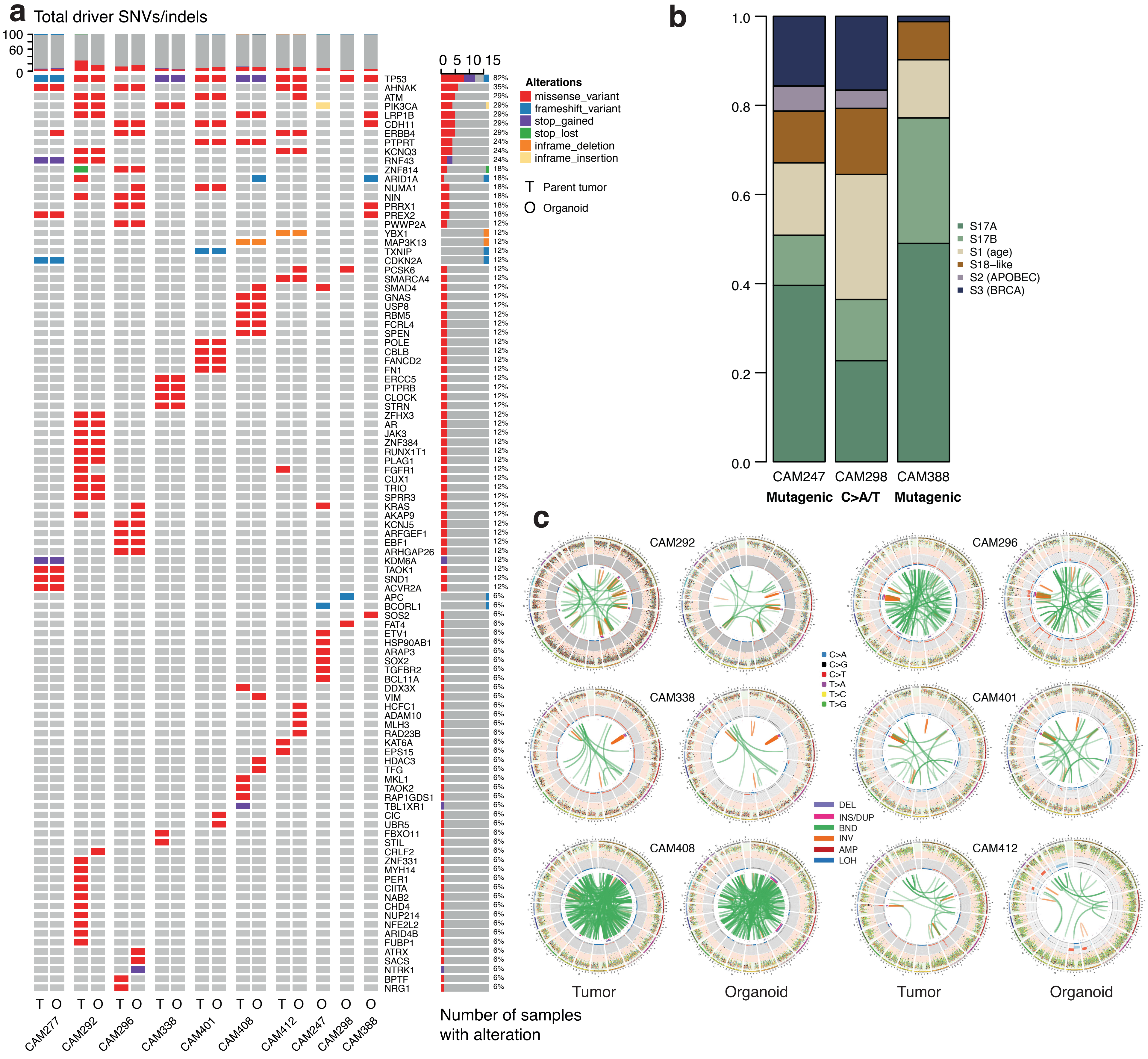

Supplementary Figure 2: Genomic characterisation of patient-matched tumor and organoid cultures

(a) Driver somatic events in the derived esophageal organoid cultures and concordance at the level of point mutations. Cancer census genes (COSMIC) affected by nonsynonymous SNVs and InDels are highlighted in matched organoid cultures and tumors (where available). (b) The mutational signature profiles for organoid cultures without patient-matched tumor WGS data. (c) Derived organoids show similar genomic landscapes to patient-matched tumors. Circos plots depicting all mutations (plotted based on inter-mutational distance), copy number changes and structural variants in the genome.

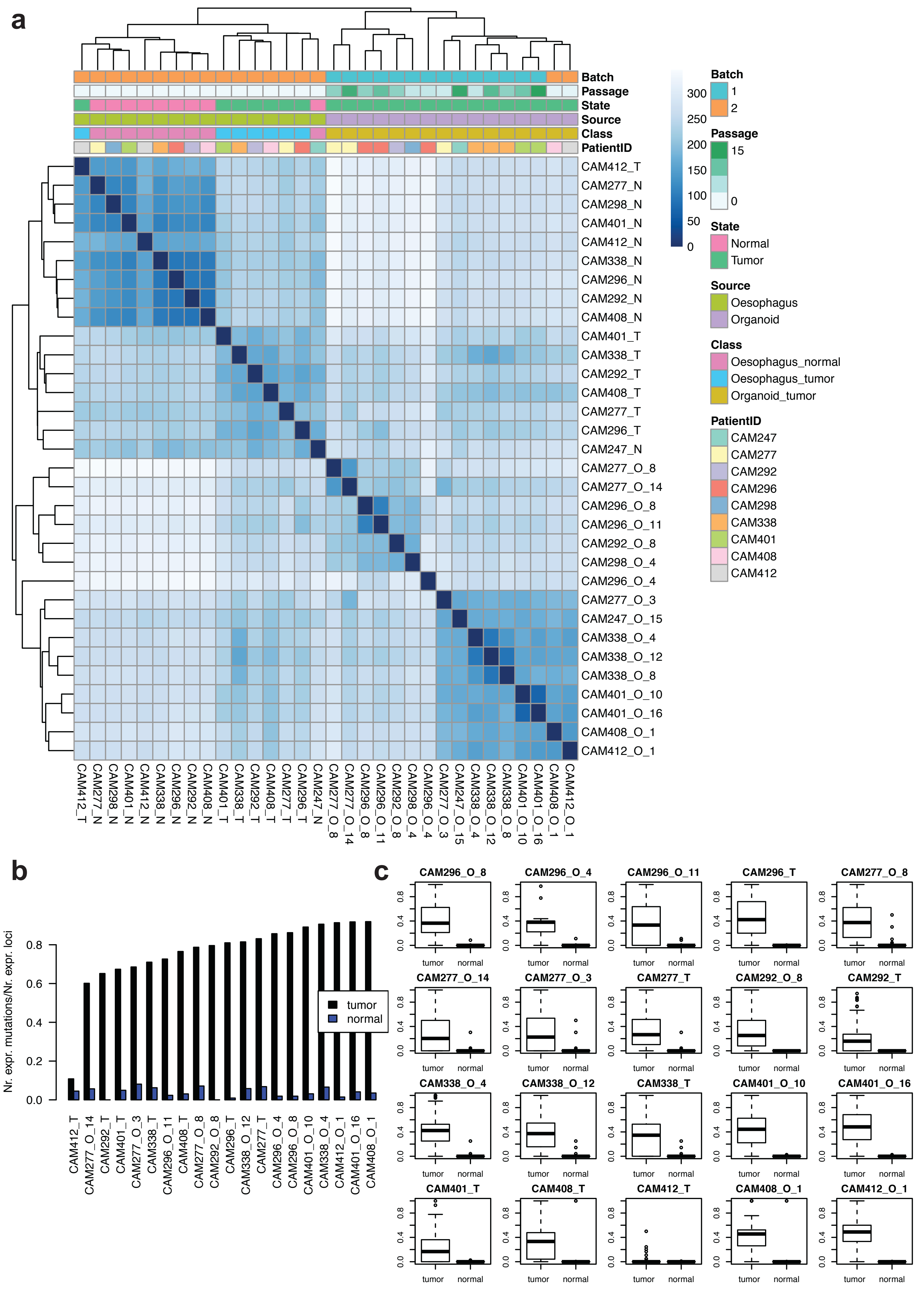

Supplementary Figure 3: Comparative expression analysis of tumors and organoids.
(a) Heatmap showing the Euclidean distance (color key) between samples based on the normalized counts of all genes. Dendrograms show the hierarchical clustering of the samples based on the complete method and the Euclidean pair-wise distance. (b) The fraction of expressed mutations out of all expressed loci in which a mutation has been detected in the WGS analysis. Mutation sets are defined by the sample indicated on the x-axis and compared to this sample itself and the associated normal sample. (c) Box and whisker plots of the variant allele fractions (y-axis) for the sets of mutations of each sample compared to the sample and the associated normal sample. The median, interquartile range and 95% confidence intervals are shown.

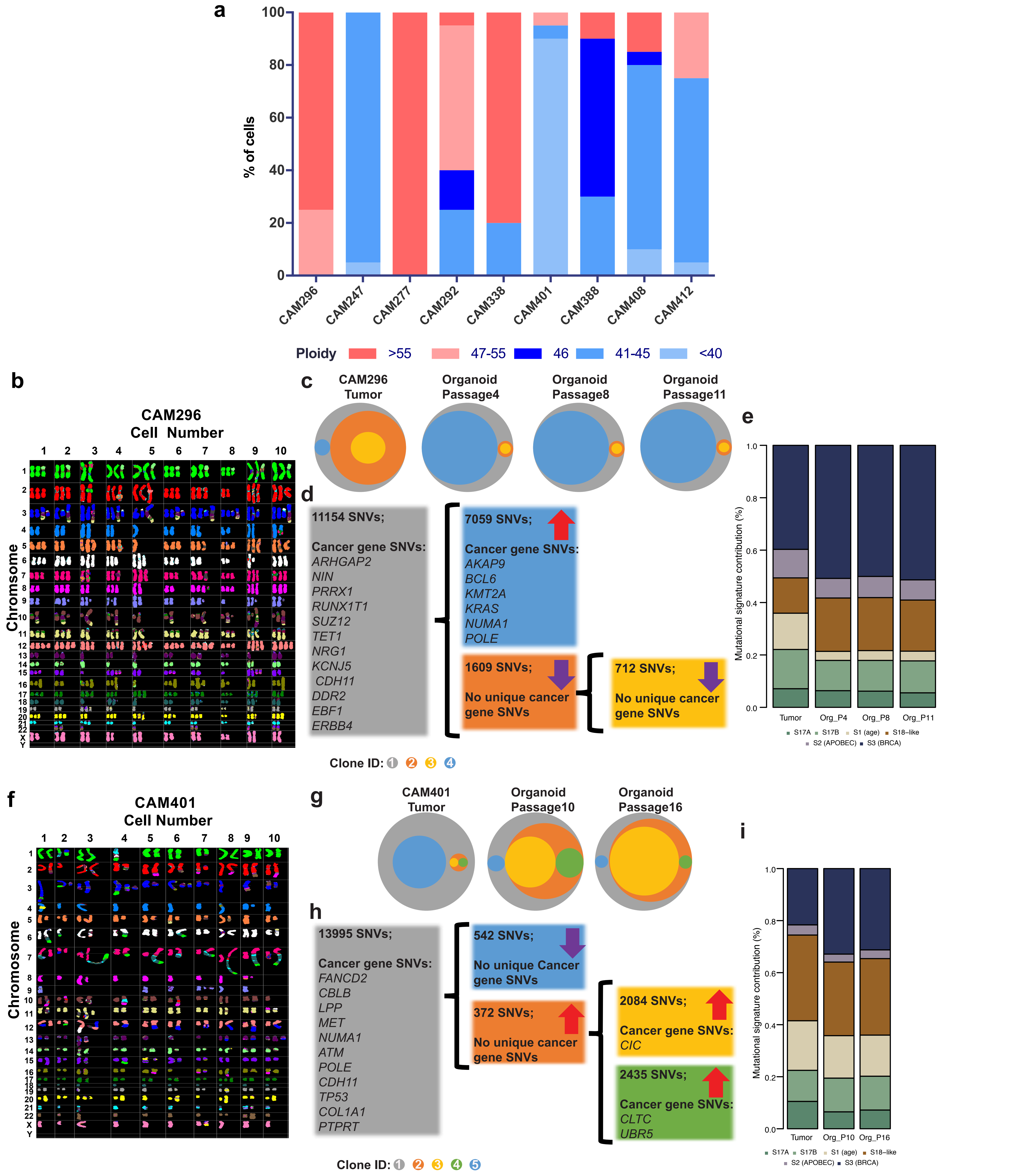

Supplementary Figure 4: EAC organoid cultures retain intra-tumor heterogeneity

(a) Ploidy analysis of organoid cultures. Results are expressed as % of cells with a given ploidy per number of metaphases counted (n = 10 cells per culture). M-FISH spectral karyotype of 10 cells from organoid culture of CAM296 (b) and CAM401 (f). CAM296 (c, d, e) and CAM401 (g, h, i) organoid cultures were continually cultured and WGS sequenced at different passages to study clonal dynamics. (c, g) Segmented Venn diagram represent clonal composition of tumor cells at each time point. The composition of each clone at different time points are listed in Supplementary Data 2. (d, h) Numbers of total mutations and mutated cancer census genes from individual subclones. (e, i) The percentage contribution of the 6 mutational signatures across successive passages. The passage number is depicted as the Org_P value.

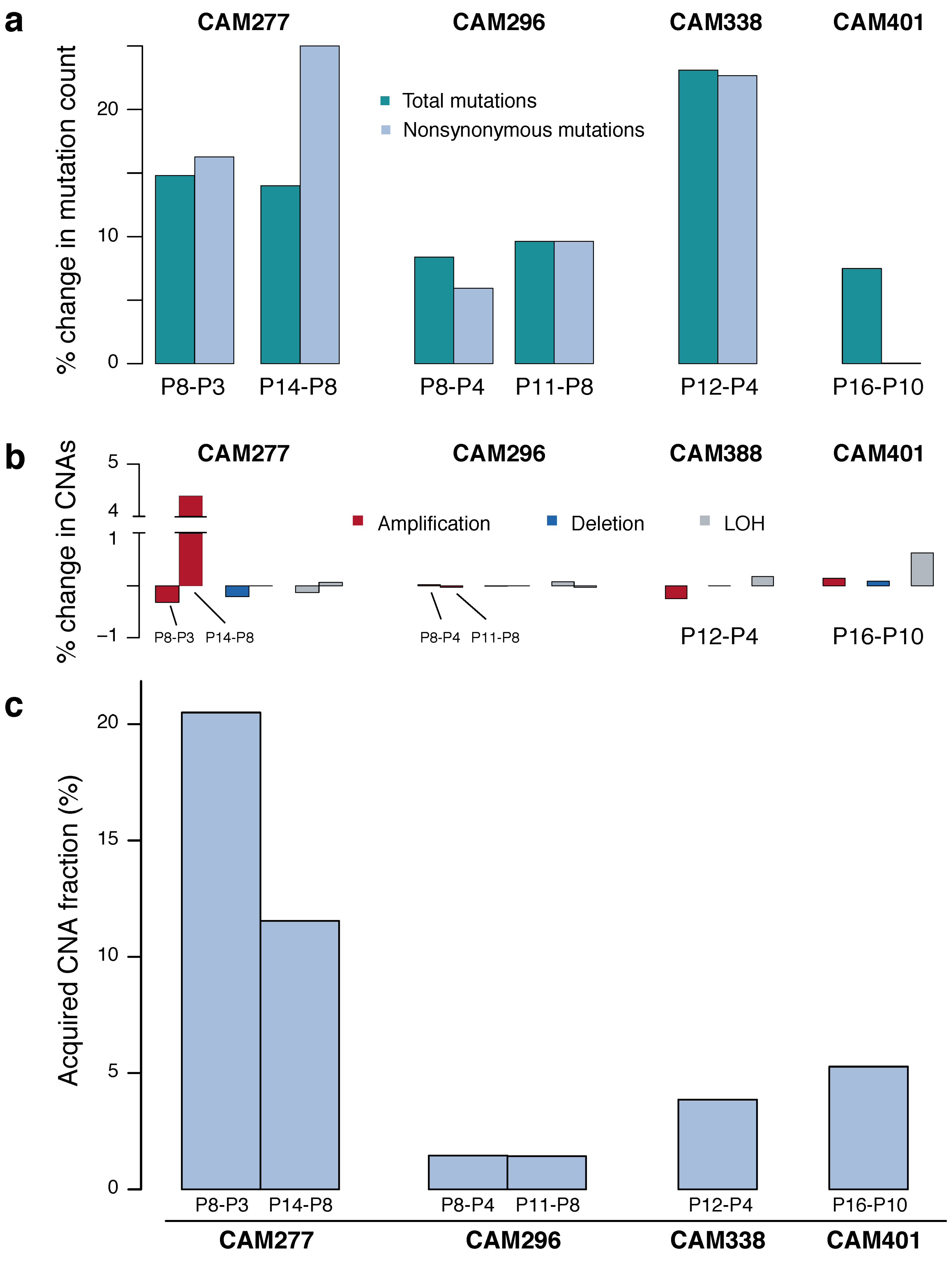

Supplementary Figure 5: Stability of genomic changes in the organoid cultures over serial passage

Changes in point mutations/InDels, copy number alterations and mutational signatures across organoid passages. (a) The percentage change in total/nonsynonymous SNVs and InDels is shown for subsequent organoid passages for the cases where data from multiple passages was available. (b) The percentage change of the genome containing amplifications, deletions or LOH, respectively, in successive organoid passages. (c) Fraction of organoid acquired copy number alteration of genome segments between passages.

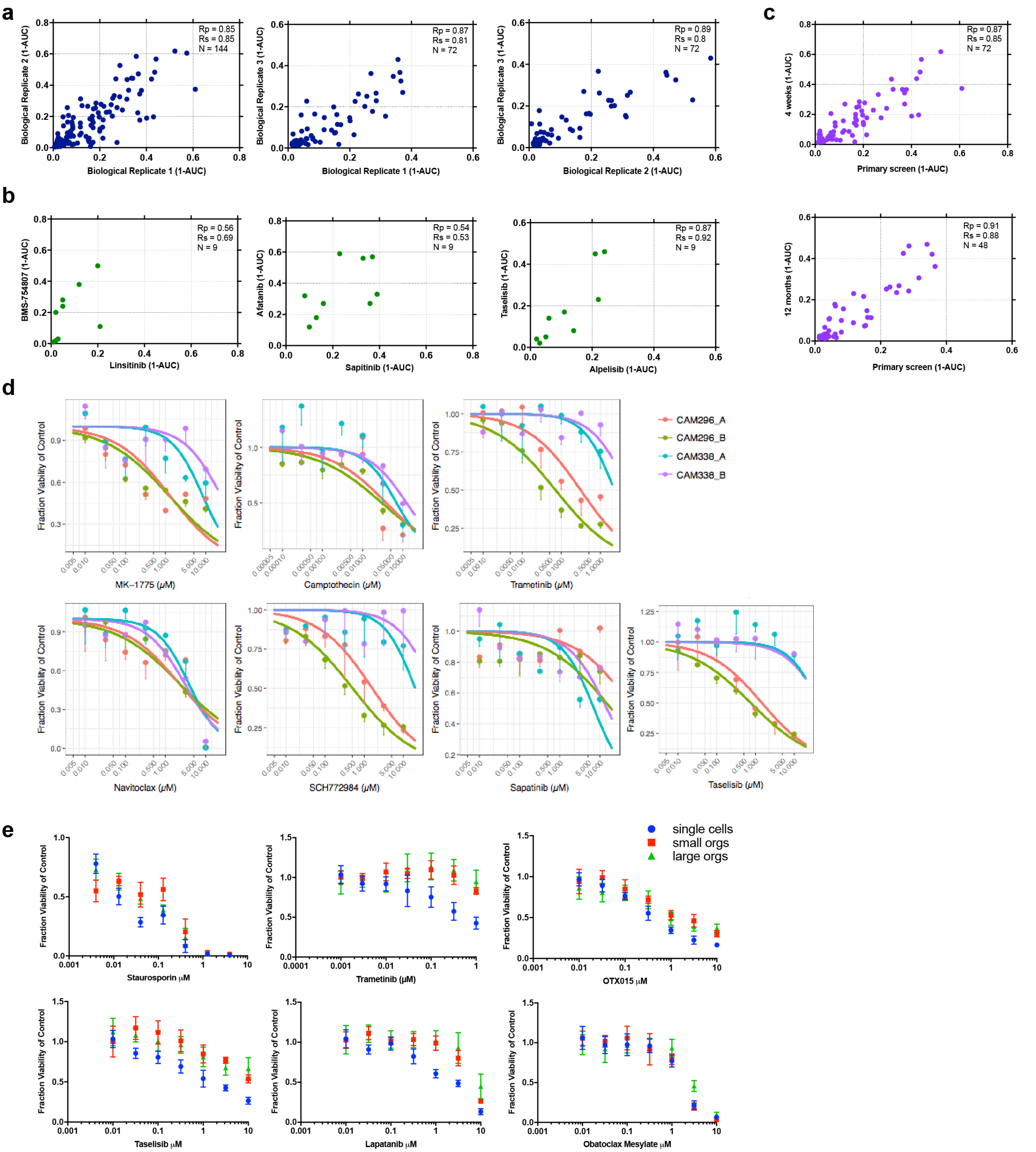

Supplementary Figure 6: Drug profiling of cancer organoids

(a) Scatterplot of (1-AUC) values for three biological replicates of drug screening data. Plots show the correlation between three biological replicates and each data point represents the (1-AUC) value for an individual organoid culture. (b) Drugs with the same nominal targets have similar activity profiles across the organoid panel. 1-AUC values are plotted for an inhibitor of IGF1R (Linsitinib and BMS-754807), EGFR-family (Afatanib and Sapitinib) and PI3K (Taselisib and Alpelisib). (c) Minimal change to drug activity (1-AUC) of 24 compounds following extended short (approximately 4 weeks) and long-term culturing (approximately 12 months) (Rs ≥ 0.85). (d) Drug sensitivity is stable following extended culturing of approximately 1 year. Dose-response curves to 6 compounds screened as technical replicates in 2 organoids approximately 1 year apart; A – primary screen, B – post 12 months in culture. (e) Consistent drug response of an organoid culture (CAM338) to 6 compounds regardless of organoids being plated as single cells, small or large organoids. All error bars are the standard deviation of triplicate measurements and 2 biological replicates.

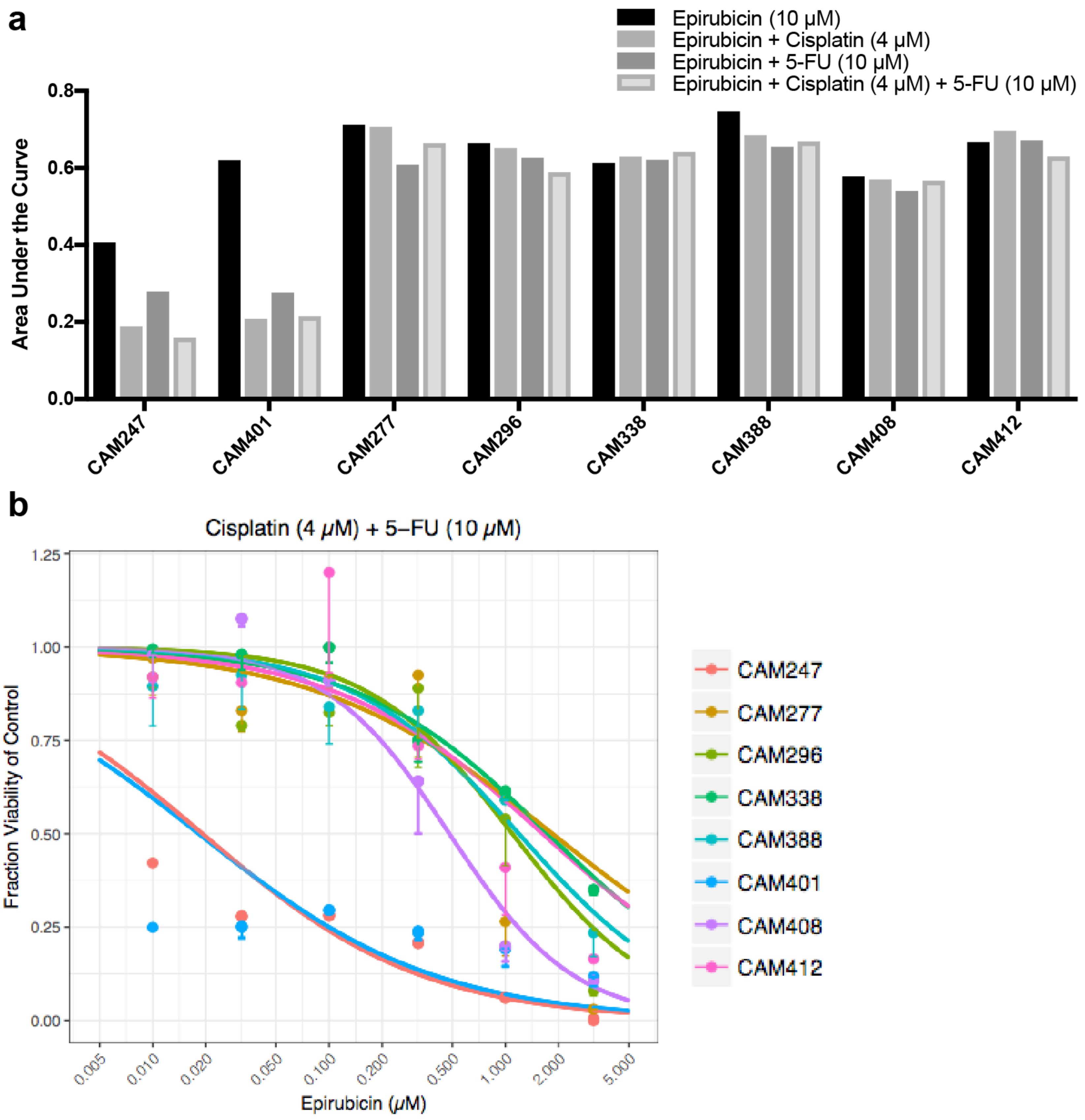

Supplementray Figure 7: Response to neoadjuvant chemotherapy response
(a) Response of each organoid model to epirubicin, cisplatin and 5-Fluorouracil (5-FU). Area under the curve values generated from technical replicates of a dose-response curve to epiruicin alone and in combination with cisplatin and 5-FU at the indicated concentrations. (b) Dose-response curves of the triple chemotherapy combination in each of the organoid models. Each experiment conducted as technical replicates and biological replicates generated for 2 models. Error bars are the standard deviation of technical replicated and biological replicates where available.

Supplementary Table 1: Summary of demographic, clinical characteristics and datasets available for EAC organoid cultures.

ID	Age	Sex	Stage	Chemo	TRG	Differentiation	Successful freeze/thaw	TP53 status	Karyotyping	Clonality	Drug Sensitivity	WGS		RNAseq		Dominant Signature	MutSigSubtype
												Primary Tumor	Organoid	Primary Tumor	Organoid		
CAM298	58	M	ypT1bN0M0	ECX	2	Well	No	mutant	No	No	No	No	Yes	No	No	S1 (age)	C>A/T dominant
CAM388	78	M	ypT2N0M0	ECX	4	Moderate to poor	Yes	mutant	Yes	No	Yes	No	Yes	No	No	S17A	Mutagenic
CAM247	67	M	ypT2N2M0	ECX	4	Moderate	Yes	wild-type	Yes	No	Yes	No	Yes	No	No	S17A	Mutagenic
CAM292	66	F	ypT3N1MX	ECX	4	Moderate to poor	Yes	mutant	Yes	No	Yes	Yes	Yes	Yes	Yes	S18-like	C>A/T dominant
CAM296	72	M	ypT3N3MX	ECF	4	Moderate to poor	Yes	wild-type	Yes	Yes	Yes	Yes	Yes	Yes	Yes	S3 (BRCA)	DDR impaired
CAM338	51	F	ypT3N1MX	ECX	5	Moderate to poor	Yes	mutant	Yes	Yes	Yes	Yes	Yes	Yes	Yes	S2 (APOBEC)	C>A/T dominant
CAM401	77	F	ypT4aN2M0	CF	5	Poor	Yes	mutant	Yes	Yes	Yes	Yes	Yes	Yes	Yes	S3 (BRCA)	DDR impaired
CAM412	52	F	cT1bN0M0	No Chemo	No Chemo	Poor	Yes	mutant	Yes	No	Yes	Yes	Yes	Yes	Yes	S17A	Mutagenic
CAM277	80	F	cT3N2M0	No Chemo	No Chemo	Poor	Yes	mutant	Yes	Yes	Yes	Yes	Yes	Yes	Yes	S18-like	C>A/T dominant
CAM408	60	M	cT1aN0M0	No Chemo	No Chemo	Moderate	Yes	mutant	Yes	No	Yes	Yes	Yes	Yes	Yes	S1 (age)	C>A/T dominant

Supplementary Table 2: EAC organoid subclonal clustering over time

Clone ID	CAM277_Tumor	CAM277_P3	CAM277_P8	CAM277_P14	No. of mutations assigned
1	102.94%	101.23%	100.43%	99.39%	16715
2	0.91%	66.71%	79.42%	87.32%	2344
3	1.92%	17.74%	26.64%	34.92%	2256
4	98.66%	45.33%	21.22%	1.19%	1495

Clone ID	CAM338_Tumor	CAM338_P4	CAM338_P12	No. of mutations assigned
1	97.91%	97.22%	92.30%	9913
2	7.84%	11.13%	22.22%	3619

Clone ID	CAM296_Tumor	CAM296_P4	CAM296_P8	CAM296_P11	No. of mutations assigned
1	98.85%	99.47%	99.18%	99.15%	11154
4	1.15%	99.82%	98.21%	99.29%	7059
2	92.16%	0.49%	0.30%	0.27%	1609
3	36.26%	0.37%	0.31%	0.37%	712

Clone ID	CAM401_Tumor	CAM401_P10	CAM401_P16	No. of mutations assigned
1	99.75%	99.30%	99.28%	13995
4	0.95%	29.10%	11.23%	2435
3	0.86%	58.08%	87.61%	2084
5	40.24%	0.45%	0.25%	542
2	1.32%	91.48%	97.88%	372

Supplementary Table 3: Sample ID and annotation for genomic sequencing data

Sample_ID	DNA_sample_ID	RNA_sample_ID	Sample_type	Sample_source
CAM247	LP6008268-DNA_F01	PR29268b	Normal	Esophagus
CAM247	PD29268a	PR29268c	Tumor	Organoid
CAM277	LP6008138-DNA_C01	PR26661g	Normal	Esophagus
CAM277	LP6008141-DNA_C01	PR26661h	Tumor	Esophagus
CAM277	PD26661a	PR26661a	Tumor	Organoid
CAM277	PD26661c	PR26661c	Tumor	Organoid
CAM277	PD26661d	PR26661e	Tumor	Organoid
CAM292	LP6008264-DNA_C03	PR29269b	Normal	Esophagus
CAM292	LP6008280-DNA_C03	PR29269d	Tumor	Esophagus
CAM292	PD29269a	PR29269a	Tumor	Organoid
CAM296	LP6008201-DNA_F01	PR26660g	Normal	Esophagus
CAM296	LP6008267-DNA_D01	PR26660h	Tumor	Esophagus
CAM296	PD26660a	PR26660a	Tumor	Organoid
CAM296	PD26660c	PR26660c	Tumor	Organoid
CAM296	PD26660d	PR26660d	Tumor	Organoid
CAM298	LP6008268-DNA_G01	PR29270b	Normal	Esophagus
CAM298	PD29270a	PR29270a	Tumor	Organoid
CAM338	LP6008264-DNA_B03	PR29271b	Normal	Esophagus
CAM338	PD29271a	PR29271a	Tumor	Organoid
CAM338	LP6008280-DNA_B03	PR29271g	Tumor	Esophagus
CAM338	PD29271c	PR29271c	Tumor	Organoid
CAM338	-	PR29271d	Tumor	Organoid
CAM388	1631_WTSI-OESO_003_b	PR37181b	Normal	Esophagus
CAM388	1631_WTSI-OESO_003_a_DNA	PR37181a	Tumor	Esophagus
CAM388	1631_WTSI-OESO_003_w6	1672_WTSI-OESO_003_w6	Tumor	Organoid
CAM401	LP6008268-DNA_E01	PR31009b	Normal	Esophagus
CAM401	LP6008269-DNA_E01	PR31009d	Tumor	Esophagus
CAM401	PD31009a	PR31009a	Tumor	Organoid
CAM401	PD31009c	PR31009c	Tumor	Organoid
CAM408	LP6008266-DNA_D01	PR37179b	Normal	Esophagus
CAM408	LP6008267-DNA_E01	PR37179a	Tumor	Esophagus
CAM408	1631_WTSI-OESO_009_w6	1672_WTSI-OESO_009_w6	Tumor	Organoid
CAM412	LP6008266-DNA_E01	PR37180b	Normal	Esophagus
CAM412	LP6008267-DNA_F01	PR37180a	Tumor	Esophagus
CAM412	1631_WTSI-OESO_040_1pre	1672_WTSI-OESO_040_1pre	Tumor	Organoid

Supplementary Methods

Summary of filters applied to single nucleotide variant (SNV) calls from Strelka.

Filter	Description
DistanceToAlignmentEndMedian	The median shortest distance of the variant position within the read to either aligned end is less than 10
DistanceToAlignmentEndMAD	The median absolute deviation of the shortest distance of the variant position within the read to either aligned end is less than 3
LowMapQual	The proportion of reads at the variant position with low mapping quality (less than 1) is greater than 10%
MapQualDiffMedian	The difference in the median mapping quality of variant reads (in the tumour) and reference reads (in the normal) is greater than 5
VariantMapQualMedian	The median mapping quality of variant reads is less than 40
VariantBaseQualMedian	The median base quality at the variant position of variant reads is less than 30
VariantAlleleCount	The number of variant-supporting reads in the tumour is less than 4
VariantAlleleCountControl	The number of variant-supporting reads in the normal is greater than 1
StrandBias	The strand bias for variant reads covering the variant position, i.e. the fraction of reads in either direction, is less than 0.02, unless the strand bias for all reads is also less than 0.02.
Repeat	The length of repetitive sequence adjacent to the variant position, where repeats can be 1-, 2-, 3-, or 4-mers, is 12 or more
SNVCluster50	The largest number of variant positions within any 50 base pair window surrounding, but excluding, the variant position is greater than 2; variant positions are those in which the number of alternate allele is supported by at least 2 reads and at least 5% of all reads covering that position.
SNVCluster100	The largest number of variant positions within any 100 base pair window surrounding, but excluding, the variant position is greater than 4; variant positions are those in which the number of alternate allele is supported by at least 2 reads and at least 5% of all reads covering that position.

Multiplex-FISH karyotyping

The organoid cultures were incubated for 3 hours with 0.1 μg/ml Karyomax Colcemid (Gibco) before being harvested and dissociated using TrypLE (Gibco). Cells were incubated with buffered hypotonic solution (0.4% KCl in 10 MM HEPES) for 8-12 minutes at 37 oC. The cells were then fixed and washed in a 6:1 (v/v) methanol:glacial fixatives and stored at -20 ^{o}C until use.

For multiplex-fluorescence in situ hybridization (M-FISH), chromosome-specific DNA libraries were generated from 5,000 copies of flow-sorted chromosomes, using GenomePlex Whole Genome Amplification (WGA2) kit (Sigma-Aldrich). Human 24-color painting probe was made following the pooling strategy (Geigl et al., 2006). Five human chromosome pools were labelled with ATTO 425-, ATTO 488-, CY3-, CY5-, and Texas Red-dUTPs (Jena Bioscience), respectively, using WGA 3 re-amplification kit (Sigma-Aldrich) and home-made dNTP mixtures optimised for the incorporation of the aforementioned labelled dUTPs by Taq polymerase. The labelled products were pooled and sonicated to achieve a size range of 200–1,000 bp, optimal for chromosome painting. The sonicated DNA sample was precipitated with ethanol together with human Cot-1 DNA (Invitrogen) and resuspended in a hybridization buffer (50% formamide, 2 × SSC, 10% dextran sulfate, 0.5 M phosphate buffer, 1 × Denhardt's solution [pH 7.4]). Metaphase preparations were dropped onto precleaned microscopic slides, followed by fixation in acetone (Sigma-Aldrich) for 10 min and dehydration through an ethanol series (70%, 90%, and 100%). Metaphase spreads on slides were denatured by immersion in an alkaline denaturation solution (0.5 M NaOH, 1.0 M NaCl) for 7-8 minutes, followed by rinsing in 1M Tris-HCl (pH 7.4) solution for 3 min, 1 × PBS for 3 min, and dehydration through a 70%, 90%, and 100% ethanol series. The M-FISH probe was denatured at 65°C for 10 min before being applied onto the denatured slides. The hybridization area was sealed with a 22 × 22-mm coverslip and rubber cement. Hybridization was carried out in a 37°C incubator for 2 nights. The post-hybridization washes included a 5-min stringent wash in 0.5 × SSC at 75°C, followed by a 5-min rinse in 2 × SSC containing 0.05% Tween20 (VWR) and a 2-min rinse in 1 × PBS, both at room temperature. Finally, slides were mounted with SlowFade Gold mounting solution containing 4′6-diamidino-2-phenylindole (Invitrogen). Images were visualized on a Zeiss AxioImager D1 fluorescent microscope equipped with narrow band-pass filters for DAPI, DEAC, FITC, CY3, TEXAS RED, and CY5 fluorescence and an ORCA-EA CCD camera (Hamamatsu). M-FISH digital images were captured using the SmartCapture software (Digital Scientific UK) and processed using the SmartType Karyotyper software (Digital Scientific UK). Approximately 20 metaphase chromosomes from each organoid culture were fully karyotyped based on M-FISH classification.

Clonality analysis

Segmental copy number information was derived for each sample using the Battenberg algorithm as previously described[1]. Briefly, the algorithm phases heterozygous SNPs with use of the 1000 genomes genotypes as a reference panel. The resulting haplotypes are corrected for occasional errors in phasing in regions with low linkage disequilibrium. After segmentation of the resulting b-allele frequency (BAF) values, t-tests are performed on the BAFs of each copy number segment to identify whether they correspond to the value resulting from a fully clonal copy number change. If not, the copy number segment is represented as a mixture of 2 different copy number states, with the fraction of cells bearing each copy number state estimated from the average BAF of the heterozygous SNPs in that segment.

Supplementary Reference

1. Nik-Zainal S, et al. The life history of 21 breast cancers. Cell 149, 994-1007 (2012).
