# Supplementary material for: Organoid cultures recapitulate esophageal adenocarcinoma heterogeneity providing a model for clonality studies and precision therapeutics
Source: Nat Commun. 2018 Jul 30;9:2983. doi: 10.1038/s41467-018-05190-9 (PMC6065407; doi:10.1038/s41467-018-05190-9)
Supplement: Supplementary file 2 — Description of Additional Supplementary Files [file 41467_2018_5190_MOESM2_ESM.pdf]

Description of Additional Supplementary Files

File Name: Supplementary Data 1
Description: List of cancer driver genes in targeted gene sequencing.

File Name: Supplementary Data 2
Description: Summary of allele frequencies for somatic variants of cancer driver genes.

File Name: Supplementary Data 3
Description: FDA approved drugs and preclinical molecularly targeted agents against key targets and pathways implicated in EAC.
